# Technical feasibility of radiomics signature analyses for improving detection of occult tonsillar cancer

**DOI:** 10.1038/s41598-020-80597-3

**Published:** 2021-01-08

**Authors:** Jeong Hoon Lee, Eun Ju Ha, Jin Roh, Su Jin Lee, Jeon Yeob Jang

**Affiliations:** 1 Division of Biomedical Informatics, Seoul National University Biomedical Informatics (SNUBI), Seoul National University College of Medicine, Seoul, 110799 Republic of Korea; 2 Department of Radiology, Ajou University School of Medicine, Wonchon-Dong, Yeongtong-Gu, Suwon, 443-380 Korea; 3 Department of Pathology, Ajou University School of Medicine, Wonchon-Dong, Yeongtong-Gu, Suwon, 443-380 Korea; 4 Department of Nuclear Medicine, Ajou University School of Medicine, Wonchon-Dong, Yeongtong-Gu, Suwon, 443-380 Korea; 5 Department of Otolaryngology, Ajou University School of Medicine, Wonchon-Dong, Yeongtong-Gu, Suwon, 443-380 Korea

**Keywords:** Diseases, Oncology

## Abstract

Diagnosis of occult palatine tonsil squamous cell carcinoma (SCC) using conventional magnetic resonance imaging (MRI) is difficult in patients with cervical nodal metastasis from an unknown primary site at presentation. We aimed to establish a radiomics approach based on MRI features extracted from the volume of interest in these patients. An Elastic Net model was developed to differentiate between normal palatine tonsils and occult palatine tonsil SCC. The diagnostic performances of the model with radiomics features extracted from T1-weighted image (WI), T2WI, contrast-enhanced T1WI, and an apparent diffusion coefficient (ADC) map had area under the receiver operating characteristic (AUROC) curve values of 0.831, 0.840, 0.781, and 0.807, respectively, for differential diagnosis. The model with features from the ADC alone showed the highest sensitivity of 90.0%, while the model with features from T1WI + T2WI + contrast-enhanced T1WI showed the highest AUROC of 0.853. The added sensitivity of the radiomics feature analysis were 34.6% over that of conventional MRI to detect occult palatine tonsil SCC. Therefore, we concluded that adding radiomics feature analysis to MRI may improve the detection sensitivity for occult palatine tonsil SCC in patients with a cervical nodal metastasis from cancer of an unknown primary site.

## Introduction

The palatine tonsil is the most common site of squamous cell carcinoma (SCC) in the oropharynx^1,2^. However, because tonsillar fossae are anatomically complex and many patients commonly have enlarged tonsils due to underlying chronic tonsillitis, identification of SCC of the palatine tonsil using physical and endoscopic examination, or conventional imaging such as computed tomography (CT) or magnetic resonance imaging (MRI) can be complicated^3–5^. Since occult palatine tonsil SCC patients commonly show a palpable neck lymphadenopathy at the initial presentation, it is difficult to differentiate them from carcinoma of unknown primary sites (CUP) of the head and neck, which makes decisions for appropriate treatment difficult for physicians as well as patients^3–5^. Currently, because more than 90% of CUPs of the head and neck reflect human papillomavirus (HPV)-associated cancers, the palatine tonsils are expected to be the most likely source of the primary cancer, and randomly directed biopsies of the tonsil and/or diagnostic tonsillectomy are commonly performed for a more focused therapy in these patients^6–9^.

Recently, several papers have reported that radiomics features derived from the volumetric analyses of a whole tumor in CT or MRI scans have shown potential for tumor detection, grading, and predicting the recurrence of head and neck cancer^10–13^. In oropharyngeal cancer, a few papers have reported the potential of radiomic features analysis (RFA) for discrimination of HPV status, tumor grading, and detection of local recurrence^11–13^. However, to date, no information exists on the potential for the early detection of occult tonsillar cancer from normal or hyperplastic lymphoid tissue. Given the histologic heterogeneity of tumor-harboring tonsillar tissue compared to normal palatine tonsils, RFA has the potential to demonstrate occult palatine tonsil SCC in patients with a neck lymph node metastasis from an unknown primary site^3^.

This retrospective study aimed to establish a technical feasibility of radiomics approach based on MRI features extracted from the volume of interest (VOI) to detect occult palatine tonsil SCC in patients with cervical nodal metastasis from a cancer of an unknown primary site.

## Results

### Comparison of radiomics features

Table 1 summarizes demographic data for overt palatine tonsil SCC, occult palatine tonsil SCC, and normal palatine tonsils in our study cohort. The mean size of the palatine tonsils in the axial, sagittal, and coronal images differed significantly among the three groups, and the values increased from normal palatine tonsils to occult palatine tonsil SCC, and to overt palatine tonsil SCC. The ANOVA analyses showed that age was significantly different (*P* = 0.006). There were no significant differences in sex among the groups (*P* = 0.226), but there was a significant difference in the status of HPV (*P* = 0.038).

**Table 1:** Patient demographic characteristics.

	Overt palatine tonsil SCC (n = 49)	Occult palatine tonsil SCC (n = 29)	Normal palatine tonsils (n = 94)	*P*-value
Age (years)	56.3 ± 8.0	63.4 ± 9.5	61.1 ± 9.4	0.006
Sex (M:F)	21:8	43:6	78:16	0.226
**Tonsil size (mm)**				
Axial	38.0 ± 8.8	31.3 ± 6.3	24.0 ± 6.2	< 0.001
Sagittal	35.3 ± 9.0	28.6 ± 6.2	21.6 ± 6.3	< 0.001
Coronal	35.3 ± 9.0	28.3 ± 6.5	21.1 ± 6.7	< 0.001
HPV status (%)	71.1 (27/38)	95.9 (25/26)	71.4 (5/7)*	0.038

Numbers for age and tonsil size are the means ± standard deviation.

*HPV* human papillomavirus; *SCC* squamous cell carcinoma.

*The percentage indicates values for a limited number of patients with a carcinoma of an unknown primary site.

**Figure 1:**
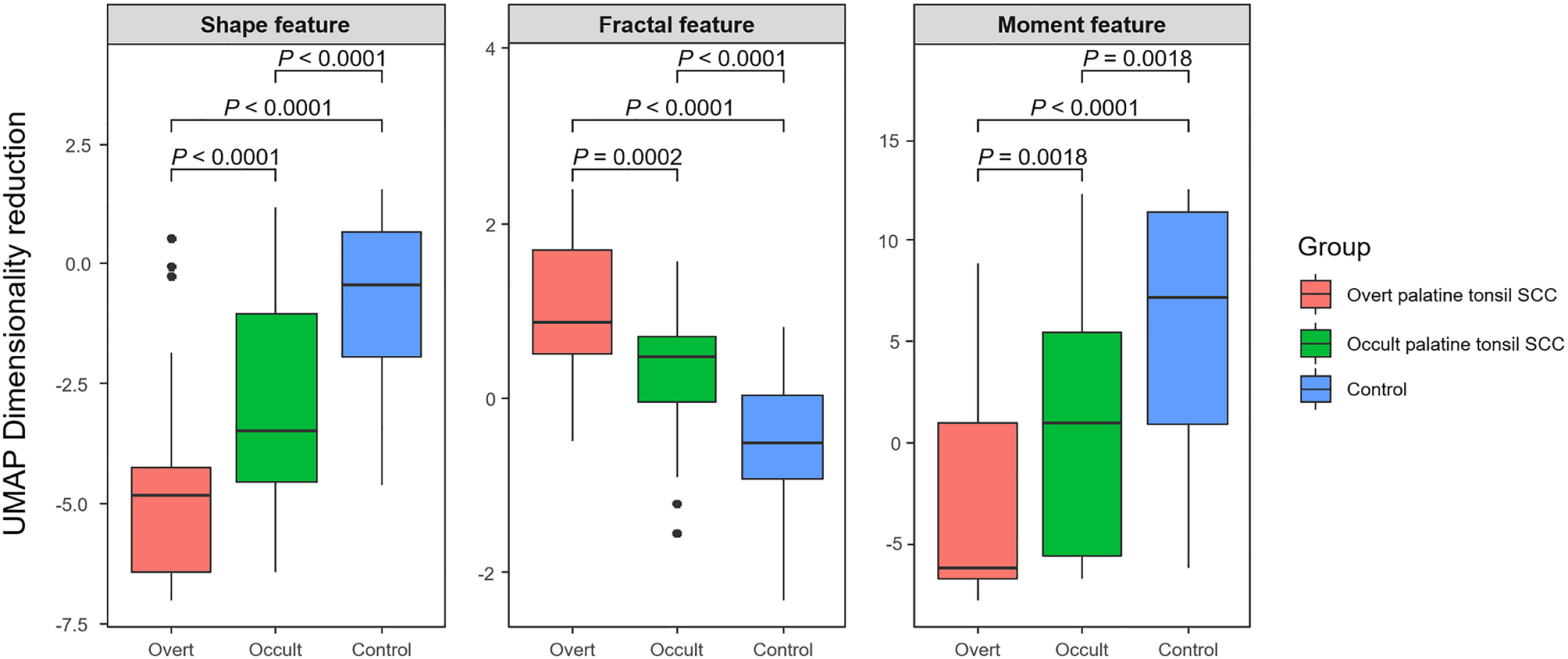
Box and whisker plots of the distribution of the representative values of shape features and fractal analyses in patients with overt palatine tonsil SCC, occult palatine tonsil SCC, and normal palatine tonsils on T2-weighted images.

**Figure 2:**
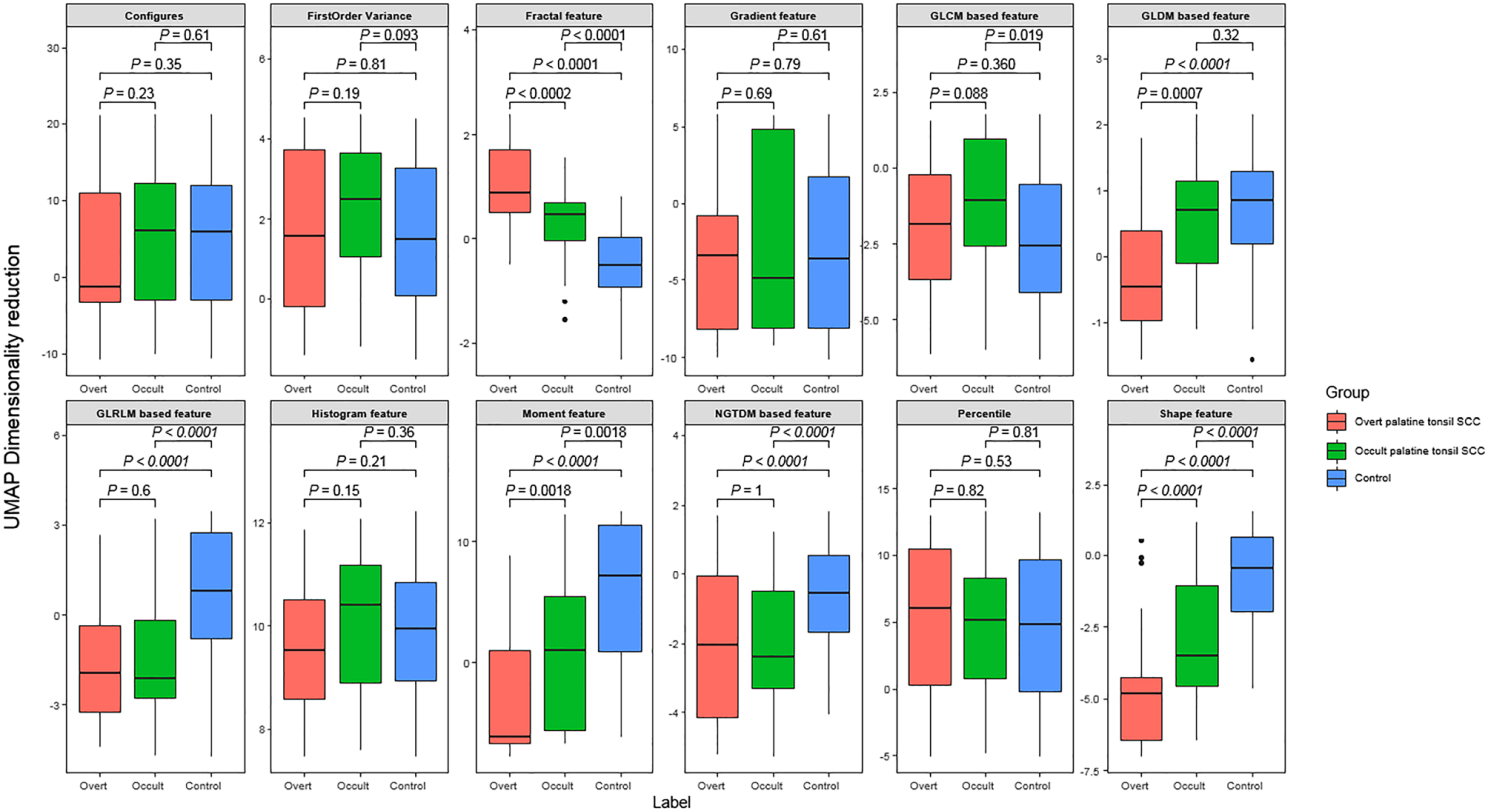
Box and whisker plots of the distribution of the representative values of radiomics features in patients with overt palatine tonsil SCC, occult palatine tonsil SCC, and normal palatine tonsils on T2-weighted images.

**Figure 3:**
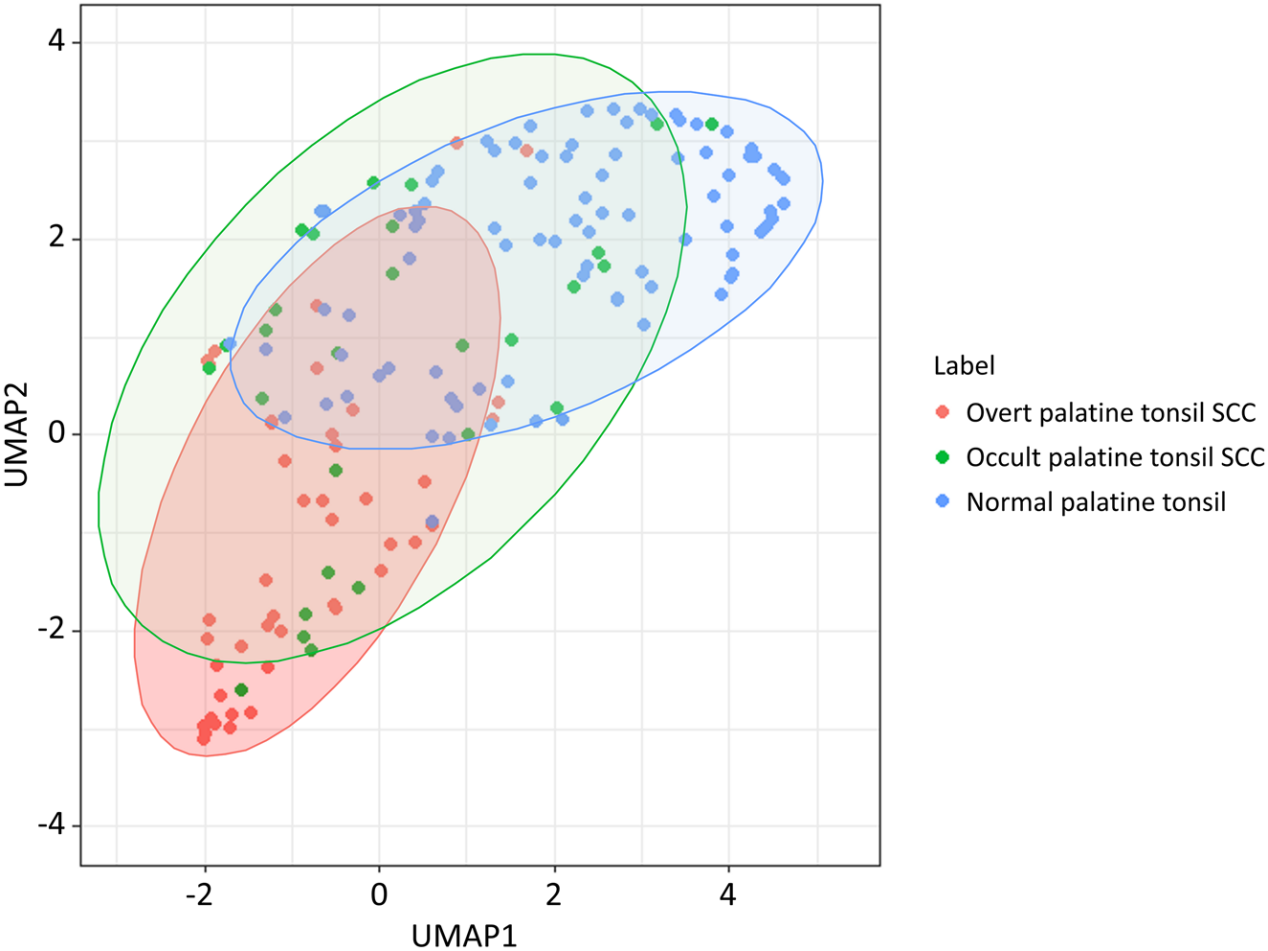
Dimensionality reduction of shape features through the Uniform Manifold Approximation and Projection algorithm^20^ (https://github.com/ropenscilabs/umapr, v0.0.0.9001).

Figure 1 shows the representative values of the radiomics features obtained through UMAP dimensional reduction. The representative values of the shape features, 3D fractal analyses, and moment features were significantly different factors among the three groups, regardless of the MRI sequences (all *P* < 0.001, respectively). The representative values of shape features, fractal analyses and moment features increased or decreased, in the order of normal palatine tonsils, occult palatine tonsil SCC, and overt palatine tonsil SCC. The UMAP dimensionality reduction results for all 12 categories are shown in the Fig. 2. This tendency was also seen in the UMAP visualization of the shape features (Fig. 3). The AUROC for the 12 feature categories to discern occult palatine tonsil SCC from normal palatine tonsils according to the MRI sequences are shown in Supplemental Table S1.

### Value of RFA in occult palatine tonsil SCC detection

The diagnostic performance of the model with whole radiomics features extracted from T1WI, T2WI, contrast-enhanced T1WI, and ADC had AUROCs of 0.831, 0.840, 0.781, and 0.807, respectively, for the differential diagnosis of occult palatine tonsil SCC from normal palatine tonsils (Table 2, Supplemental Table S2). In terms of sensitivity, the best performing model with features from ADC alone showed the highest sensitivity of up to 90.0%. The model with features from T1WI + T2WI + contrast-enhanced T1WI had the highest AUROC of up to 0.853 (Table 3, Supplemental Table S2).

**Table 2:** Diagnostic performance of RFA to discern occult palatine tonsil SCC from normal palatine tonsils according to the MRI sequences.

MRI sequences	AUROC	Sensitivity (%)	Specificity (%)
T1WI	0.831	81.4	89.5
T2WI	0.840	86.5	82.4
Contrast-enhanced T1WI	0.781	83.2	80.2
ADC	0.807	90.0	77.9

*ADC* apparent diffusion coefficient; *AUROC* area under the receiver operating curve; *T1WI* T1-weighted image; *T2WI* T2-weighted image.

**Table 3:** Diagnostic performance of radiomics features to discern occult palatine tonsil SCC from normal palatine tonsils according to a combination of MRI sequences.

MRI sequence	AUROC	Sensitivity (%)	Specificity (%)
ADC	0.807	90.0	77.9
T1WI + T2WI	0.810	89.7	75.4
T1WI + contrast-enhanced T1WI	0.765	81.4	79.8
T2WI + contrast-enhanced T1WI	0.843	84.8	85.3
T1WI + T2WI + contrast-enhanced T1WI	0.853	81.4	87.4

*ADC* apparent diffusion coefficient; *AUROC* area under the receiver operating characteristics; *T1WI* T1-weighted image; *T2WI* T2-weighted image.

### Additional value of radiomics over conventional MRI or ^18^F-FDG PET/CT

Table 4 shows the diagnostic performance of the different imaging modalities to detect occult palatine tonsil SCC among patients with cervical lymph node metastasis from CUP at presentation. The sensitivity and accuracy of conventional MRI alone were 41.4% and 51.4%, respectively, and those of ^18^F-FDG PET/CT were 79.3% and 75.7%, respectively. The SUVmax of the occult palatine tonsil SCC was 9.3 ± 3.4 (range 2.5–16.2), and that of the contralateral tonsil was 5.5 ± 1.7 (range 2.4–10.6). The mean difference in the SUVmax between the two (occult palatine tonsil SCC—contralateral normal palatine tonsil) was 3.8 ± 3.5 (range − 4.5 to 11.0). The added value of the RFA of ADC was 34.6% for sensitivity and 28.9% for accuracy over conventional MRI alone. This indicates comparable sensitivity and accuracy compared to ^18^F-FDG PET/CT.

**Table 4:** Diagnostic performance to detect occult palatine tonsil SCC using different imaging modalities.

Modality	Sensitivity	Specificity	Accuracy	PPV	NPV
Radiomics feature (ADC)	76.0	80.0	80.3	94.1	57.7
MRI	41.4	87.5	51.4	92.3	29.2
^18^F-FDG PET/CT	79.3	62.5	75.7	88.4	53.8

Numbers for diagnostic performance are percentages.

*ADC* apparent diffusion coefficient; *PPV* positive predictive value; *NPV* negative predictive value.

## Discussion

Our study demonstrated that a radiomics approach based on MRI features extracted from a VOI of the palatine tonsil has the potential to differentiate occult palatine tonsil SCC from normal palatine tonsils in patients with cervical nodal metastasis from a CUP. The added sensitivity of RFA for detecting occult palatine tonsil SCC was 34.6% over conventional MRI and it was comparable to ^18^F-FDG PET/CT.

Despite recent advances in diagnostic tools for use in head and neck cancers, it is common to encounter patients who present with cervical lymph node metastasis without an apparent primary site following thorough clinical and radiological examinations^6,14^. In previous studies, SCC was the most common histologic type, accounting for 78% of cases. However, the treatment strategies have been controversial, until now^6,8,9^. Although radiation therapy with or without neck dissection, including irradiation of all potential mucosal disease sites, has been effective, it causes substantial morbidity and side effects^6,14^. Therefore, efforts should be made to find the primary tumor, as this allows a more focused therapy, with less morbidity, and possibly a better outcome^9^. In this respect, a randomly directed biopsy and/or diagnostic tonsillectomy is common, as up to 25% of primary tumors can be detected in this way whereas some have a delayed diagnosis of the primary tumor during the follow-up period^9^.

More recently, research based on medical imaging informatics has greatly improved. Radiomics, a data mining approach that extracts high-dimensional data in the form of a multitude of features from clinical images to build machine-learning or statistical models, has been applied to various imaging modalities to answer relevant clinical questions^10^. In the field of head and neck cancer radiomics, classification and survival regression models have been applied to predict molecular markers and identify genomic signatures for the diagnostic differentiation of suspected tissues, survival prognostication, and to predict treatment responses^11–13^. In our study, we developed models using radiological images and integrated quantitative radiomics features to arrive at a better clinical decision and treatment planning for head and neck cancer. We focused on the potential of a radiomics approach to differentiate occult palatine tonsil SCC from normal palatine tonsil, based on the histologic heterogeneity of tumor-harboring tonsillar tissue compared to normal palatine tonsils. Representative values of shape features, 3D fractal analyses, and moment features showed significant differences among normal palatine tonsils and occult and overt palatine tonsil SCC, regardless of the MRI sequences. Based on these results, the model with the radiomics features extracted from an ADC map showed the highest sensitivity of up to 90.0%. These results are similar to those of Choi et al., which showed the potential for histogram analyses of ADC for differential diagnosis of occult palatine tonsil SCC. Adding histogram analyses of ADC to conventional MRI showed the potential to improve the detection sensitivity up to 52.6% (from 26.3% to 78.9%) in their study^3^.

The application of advanced diagnostic methods such as ^18^F-FDG PET-CT can be useful for differential diagnosis in patients with CUP^15,16^. A previous study that included data from 302 patients with occult primary head and neck tumors found that FDG PET detected 24.5% more histologically proven primary lesions compared to conventional assessment methods^17^. However, because the range of physiological FDG uptake in normal palatine tonsil varies considerably, establishing a cut-off threshold to distinguish between normal palatine tonsil uptakes from occult palatine tonsil SCC is not easy^15,18^. In addition, FDG PET had a false positive rate of 39.3% for detecting occult palatine tonsil primary cancers^17^. In our study, the mean differences in the SUVmax between the occult palatine tonsil SCC and the contralateral normal palatine tonsil also varied from − 4.5 to 11.0. Because a subtle asymmetric difference between the bilateral tonsils without tonsillar malignancy is also common in practice, clinical decisions in patients with CUP are sometimes very difficult. In this study, adding the RFA over conventional MRI alone showed the potential for improving sensitivity, which was comparable to ^18^F-FDG PET-CT. Further research in a larger population sample is needed based on these results.

There were some limitations to our study. First, we only included 29 patients with occult palatine tonsil SCC and six patients who had an MRI in another institution were excluded. Since an external validation cohort is lacking in this study, further prospective studies with a larger population are necessary to validate of our study results and to achieve reproducibility and generalizability. Second, there may be a technical challenge in delineating tonsil boundaries when drawing the regions of interest and it may produce segmentation errors in tumor volumes and their associated radiomic features^3^. Stability analysis of MRI radiomic features in palatine tonsils are required in the future.

In conclusion, occult palatine tonsil SCC may be differentiated from normal palatine tonsils using RFA in patients with cervical nodal metastases from CUP. A radiomics approach has the potential to improve targeted treatment and reduce morbidity.

## Methods

### Study population

Our study protocol was reviewed and approved by the Ajou University hospital Institutional Review Board. Ethics committees/Institutional Review Board (AJIRB-MED-MDB-18-508) waived the need for you to obtain informed consent because of the retrospective nature of the study. All methods were performed in accordance with the relevant guidelines and regulations. We reviewed the medical records of patients at our institution between May 2010 and April 2020 that had cervical nodal metastasis from CUP at presentation and which was finally confirmed as palatine tonsillar SCC. We enrolled the patients who met the following criteria: a histologically proven metastatic SCC in cervical lymph node at clinical presentation, negative or equivocal findings of a contrast-enhanced neck CT scan, a pretreatment neck MRI examination, and a pathologically proven palatine tonsil SCC after a tonsillectomy or biopsy. A total of 39 patients with occult palatine tonsil SCC that had cervical nodal metastasis at presentation were identified. After the exclusion of 10 patients who did not undergo pretreatment neck MRI or an MRI outside of the hospital (n = 6), or who had a severe dental or motion artifact on the MRI (n = 4), 29 patients were finally enrolled (mean age, 56.3 years; age range, 32–74 years). Tumor grades were determined by an experienced pathologist (J. R.: 10 years of clinical experience) to be well-differentiated (n = 2), moderately differentiated (n = 18), or poorly differentiated (n = 9).

For comparison, we enrolled 49 patients with an overt palatine tonsil SCC (mean age, 63.4 years; age range, 44–82 years) as positive control subjects as follows: pathologically proven palatine tonsil SCC, evidence of an overt palatine tonsil SCC on a contrast-enhanced neck CT, and a pretreatment neck MRI at our hospital. Bilateral palatine tonsils from eight patients with CUP of head and neck (mean age, 54.5 years; age range, 36–74 years) and the contralateral palatine tonsils of 78 patients with an occult or overt palatine tonsil SCC were included as negative control subjects with a histopathologic confirmation^3^.

### Imaging Techniques

All MR examinations were performed with 1.5 or 3T MRI units (Signa HDxt or Discovery MR 750; GE Healthcare Systems, Illinois, USA). In all of the patients, the protocol included axial T2-weighted fast spin-echo images with fat suppression (4 mm slice thickness with 0.4 mm slice spacing), axial T1-weighted fast spin-echo images (4 mm slice thickness with 0.4 mm slice spacing), and contrast-enhanced axial T1-weighted images (WI) following a bolus injection of 0.1 mmol per kilogram of body weight of gadoteridol (ProHance; Bracco, Milan, Italy). All T1WI and T2WI were acquired with 30 imaging sections and a field of view of 200 (anterior to posterior) × 200 (right to left) × 120 (feet to head) mm. Diffusion-weighted imaging was performed before the contrast-enhanced T1WI; 30 fat-suppressed diffusion-weighted images of the head and neck were acquired in the axial plane using a spin-echo single-shot echo-planar image sequence (section thickness 4 mm/gap 0.4 mm; field of view, 230 (anterior to posterior) × 230 (right to left) × 120 (feet to head) mm; acquisition matrix 112 × 112; reconstruction matrix 256 × 256; b values of 0 and 1000 s/mm^2^) in the axial plane from the skull base to the hyoid bone level. The diffusion gradients were applied in three orthogonal directions (x, y, and z). Axial trace apparent diffusion coefficient (ADC) maps were generated for all of the obtained images with the manufacturer’s software included in the MR unit using the b values of 0 and 1000 s/mm^2^^3^.

### Imaging processing and analyses

**Figure 4:**
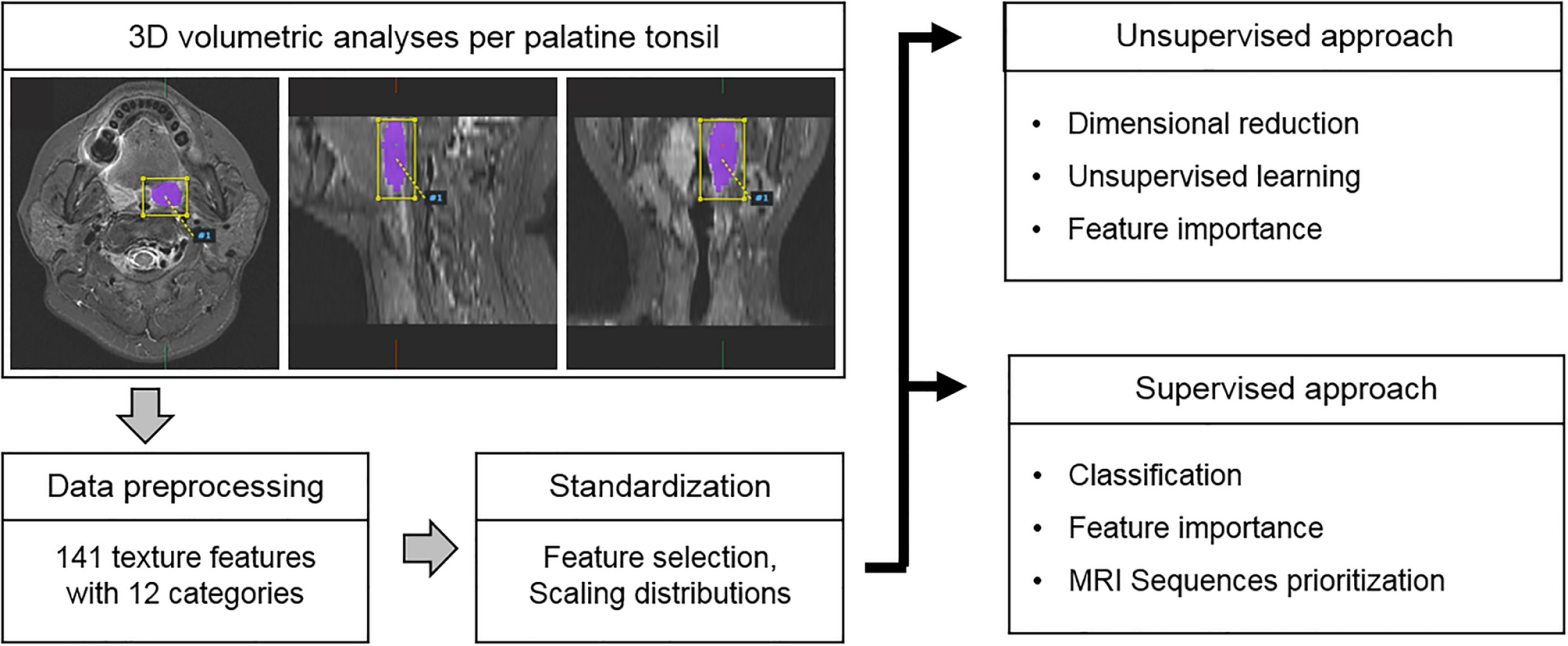
Workflow scheme of this study. Flow diagrams show the process from a VOI segmentation to model evaluation.

RFA was performed using Pyradiomics in A-VIEW Research 1.0v (Coreline Soft; Seoul, Korea; https://www.corelinesoft.com/aview-research-2/)^19^. The T1WI, T2WI, contrast-enhanced T1WI, and ADC maps of all patients were exported to the software. Three-dimensional VOIs per palatine tonsil were semi-automatically created by the software, and an experienced radiologist (E.J.H.: 15 years of clinical experience) modified the VOIs on each axial images. For each VOI, 141 texture features with 12 categories were computed, including shape features (26 features), first-order statistics features (19 features), and histogram/percentile/gradient features (17 features). Second-order statistics features were derived from the gray-level co-occurrence matrix (GLCM, 24 features), gray-level dependence matrix (GLDM, 14 features), gray-level run length matrix (GRLM, 16 features), gray-level size zone matrix (GSZM, 16 features), and neighborhood gray-tone difference matrix (NGTDM, 5 features). Higher-order features included fractal analyses (1 feature) and moment features (3 features). All features were transformed to the same scale through Z-score normalization. There was no further manipulation in a pre-processing step before the radiomics feature extraction. The study workflow is detailed in Fig. 4.

To assess the value of adding RFA to conventional MRI and 18F-fluorodeoxyglucose (FDG) positron emission tomography (PET)/CT for differentiating normal palatine tonsils from occult palatine tonsil SCC, one radiologist (E.J.H.) reviewed conventional MRIs of 29 patients with occult palatine tonsil SCC and 8 patients with CUP, including T1WI, T2WI, and contrast-enhanced T1WI, and the radiologist was blinded to all other data including the final histologic diagnoses. In the MRIs, SCC of the palatine tonsil was defined as a mass that could be discriminated from the surrounding tonsillar tissue by its signal intensity or the degree of enhancement. Tonsillar asymmetry was also considered positive for the presence of palatine tonsil SCC^3^. One nuclear medicine physician (S.J.L.: 16 years of clinical experience) reviewed 18F-FDG PET/CT images, and asymmetric hypermetabolism on the 18F-FDG PET/CT images was considered to indicate a positive palatine tonsil SCC finding. The maximum standardized uptake values (SUVmax) of the bilateral palatine tonsils were recorded as reference values.

### Statistical analyses

Statistical analyses were performed using R v. 3.6.3. (R Foundation for Statistical Computing, Vienna, Austria). One-way analysis of variance (ANOVA) was used to compare the demographic characteristics among the groups. A Wilcoxon test was used to compare the quantitative texture feature categories among the groups. To obtain representative values of the feature categories, a nonlinear dimension reduction algorithm, Uniform Manifold Approximation and Projection (UMAP), was applied, except for the fractal analyses, which comprised only one feature^20^. The discriminative power of the representative value of each category was evaluated through the geometric means of the p-value through group-wise comparisons.

Elastic Net regularization for generalized linear models is the linear combination of lasso and ridge regularization methods^21^. An Elastic Net model was developed to differentiate between normal palatine tonsils and occult palatine tonsil SCC. We constructed area under the receiver operating characteristic (AUROC) curves to determine the best predictive model and threshold values from the radiomics features. The best predictive model was selected based on an AUROC of fivefold cross-validation, and the alpha parameter of the Elastic Net was set to 0.5. In each fivefold cross-validation, the test set was predicted through the penalty parameter with the minimum mean cross-validated error in the training set. A *P* value < 0.05 was considered statistically significant.

## Supplementary Information

Supplementary Information.
