# Supplementary material for: Technical feasibility of radiomics signature analyses for improving detection of occult tonsillar cancer
Source: Sci Rep. 2021 Jan 8;11:192. doi: 10.1038/s41598-020-80597-3 (PMC7794329; doi:10.1038/s41598-020-80597-3)
Supplement: Supplementary file 1 — Supplementary Information. [file 41598_2020_80597_MOESM1_ESM.doc]

Technical Feasibility of Radiomics Signature Analyses for Improving Detection of Occult Tonsillar Cancer

**Supplemental Table 1.** The area under the receiver operating characteristic curve (AUROC) for the 12 feature categories to discern occult palatine tonsil SCC from normal palatine tonsils according to the MRI sequences

|  | T1WI | T2WI | Contrast-enhanced T1WI | ADC | Mean |
| --- | --- | --- | --- | --- | --- |
| Shape feature | 0.839 | 0.820 | 0.773 | 0.809 | 0.810 |
| Histogram feature | 0.798 | 0.835 | 0.764 | 0.831 | 0.807 |
| FirstOrder Variance | 0.781 | 0.791 | 0.760 | 0.812 | 0.786 |
| GLDM- based features | 0.783 | 0.832 | 0.709 | 0.790 | 0.779 |
| Fractal feature | 0.805 | 0.766 | 0.742 | 0.787 | 0.775 |
| NGTDM- based features | 0.761 | 0.813 | 0.714 | 0.802 | 0.773 |
| Moment feature | 0.770 | 0.814 | 0.702 | 0.771 | 0.764 |
| GLRLM- based features | 0.764 | 0.734 | 0.720 | 0.806 | 0.756 |
| GLCM- based features | 0.634 | 0.673 | 0.596 | 0.773 | 0.669 |
| Gradient feature | 0.649 | 0.631 | 0.470 | 0.643 | 0.598 |
| Percentile | 0.587 | 0.590 | 0.542 | 0.561 | 0.570 |
| **Whole Features** | **0.803** | **0.834** | **0.814** | **0.775** | **0.807** |

ADC: apparent diffusion coefficient; GLCM: gray level co-occurrence matrix; GLDM: gray level dependence matrix; GLRLM: gray level run-length matrix; NGTDM: neighborhood gray-tone difference matrix; T1WI: T1-weighted image; T2WI: T2-weighted image

**Supplemental Table 2.** Detailed diagnostic performance of RFA to discern occult palatine tonsil SCC from normal palatine tonsils according to the MRI sequences

|  | Number | Sensitivity (%) | Specificity (%) | True positive | True negative | False positive | False negative |
| --- | --- | --- | --- | --- | --- | --- | --- |
| T1WI | 123 | 81.4 | 89.5 | 24 | 84 | 5 | 10 |
| T2WI | 123 | 86.5 | 82.4 | 25 | 77 | 4 | 17 |
| Contrast-enhanced T1WI | 123 | 83.2 | 80.2 | 24 | 75 | 5 | 19 |
| ADC | 102 | 90.0 | 77.9 | 23 | 60 | 3 | 17 |
| T1WI + T2WI | 123 | 89.7 | 75.4 | 26 | 71 | 3 | 23 |
| T1WI + contrast-enhanced T1WI | 123 | 81.4 | 79.8 | 24 | 75 | 5 | 19 |
| T2WI + contrast-enhanced T1WI | 123 | 84.8 | 85.3 | 25 | 80 | 4 | 14 |
| T1WI + T2WI + contrast-enhanced T1WI | 123 | 81.4 | 87.4 | 24 | 82 | 5 | 12 |

ADC: apparent diffusion coefficient; AUROC, area under the receiver operating curve; T1WI: T1-weighted image; T2WI: T2-weighted image
